# Taking it up a NOTCH: a novel subgroup of ACC is identified

**DOI:** 10.18632/oncotarget.20879

**Published:** 2017-09-14

**Authors:** Renata Ferrarotto, John V. Heymach

**Affiliations:** Renata Ferrarotto: Thoracic and Head and Neck Medical Oncology Department, University of Texas MD Anderson Cancer Center, Houston, TX, USA

**Keywords:** adenoid cystic carcinoma, NOTCH1, targeted therapy, salivary gland cancer, Notch inhibitors

Adenoid Cystic Carcinoma (ACC) is a neoplasm of the secretory glands and represents the second most common malignancy of the salivary gland. The majority of patients present with localized disease, however, approximately half will recur distantly in spite of aggressive curative intent treatment. While ACC typically recurs in the lungs and has an indolent disease course, approximately 15% of patients exhibit a more aggressive disease phenotype [1]. *MYB or MYBL1-NFIB* translocation is present in the vast majority of ACCs and it leads to myb protein overexpression, however, targeting transcription factors is clinically challenging [1]. Exome-sequencing of ACC samples revealed common alterations in Notch-pathway genes, particularly *NOTCH1* mutations in patients with metastatic disease [2]. *NOTCH1* is involved in a well-conserved developmental pathway and can function as either an oncogene or a tumor suppressor, depending on the cellular context. Pathway dysregulation can lead to many cancer-relevant cellular functions such as proliferation, stemness, epithelial-mesenchymal transition, genomic instability, and angiogenesis [3]. Notch signaling is usually initiated by receptor-ligand interaction, which ultimately leads to receptor cleavage by the gamma-secretase complex that frees the Notch intracellular domain to enter the nucleus and form a transcriptional activation complex [3]. In ACC, most mutations in *NOTCH1* are activating and result in loss of the C-terminal PEST degron domain, resulting in reduced degradation of the NOTCH1 protein. Less frequently, point substitutions and in-frame insertions/deletions disrupting the negative regulatory region (NRR) are identified, which leads to ligand-independent receptor proteolysis [2]. In our analyses, while *NOTCH1* activating mutations was seen in 14% of ACC patients, pathway activation was detected in 56% by immunohistochemistry using a validated, specific antibody against the cleaved notch1 intracellular domain (NICD1), suggesting there are mechanisms other than *NOTCH1* mutation that lead to pathway activation. Indeed, other mutations predicted to activate the Notch pathway, such as loss-of-function mutations in *SPEN*, a negative transcriptional regulator of Notch, were identified in ACC and tend to co-occur with *NOTCH1* activating mutations [2]. There is also evidence that *NOTCH1* is a putative myb target in ACC and therefore *MYB-NFIB* translocations could lead to Notch pathway activation [4]. Using detailed histologic and clinical annotation, we demonstrated that *NOTCH1* mutations significantly correlate with solid histology, advanced disease stage at diagnosis, higher incidence of liver and bone metastasis, and shorter relapse-free and overall survival [2]. This association between Notch pathway activation and pro-metastatic phenotype is not exclusively in ACC and has been reported in other tumors such as breast cancer and chronic lymphocytic leukemia (CLL) [2].

To test the hypothesis that Notch1 is a therapeutic target in a subset of ACC, we and others have screened genotyped ACC patient-derived xenograft (PDX) models with Notch inhibitors and observed significant tumor growth inhibition exclusively in the ACCX9 *NOTCH1* mutant model [2, 5]. We then identified an index ACC patient with aggressive disease that harbored at least two *NOTCH1* activating mutations (in the NRR and PEST domains), who achieved a partial response when treated with brontictuzumab, a specific monoclonal antibody targeting Notch1 [2]. Clinical response to gamma-secretase inhibitors was also reported in another *NOTCH1* mutant ACC patient, supporting the oncogenic role of *NOTCH1* and its inhibition as an attractive therapeutic strategy in ACC [5, 6]. While these responses are encouraging, the clinical development of Notch inhibitors has been challenging. This class of agents can cause significant on-target toxicity, mainly diarrhea, which may limit the achievement of therapeutically relevant doses or continuous Notch inhibition. Moreover, the mechanism of action of the currently available drugs might not be able to successfully inhibit the pathway depending on its mode of activation. Thus far, most ACC patients that participated in phase I trials of Notch inhibitors experienced disease stabilization rather than objective response, with the caveat that these studies did not select patients based on their *NOTCH1* mutation status, highlighting the importance of patient selection [6, 7]. Additionally, at least in the reported index case [2], resistance developed early on upon Notch inhibition suggesting activation of alternative signaling pathways upon Notch inhibition that lead to tumor growth and/or immune evasion, or that other genetic events, such as myb translocations, cooperates with *NOTCH1* mutations to promote oncogenesis.

While many questions remain, transcriptomic and proteomic characterization of *NOTCH1* mutant ACCs is ongoing and will shed light into the biology of this aggressive patient subgroup and guide future studies utilizing rational drug combinations. Furthermore, new in class pan-NOTCH inhibitors that directly target the Notch transcriptional activation complex have shown encouraging activity in gamma-secretase inhibitor refractory Notch activated triple negative breast cancer mouse models and will soon be tested in a first-in-human trial [8]. Clinical studies testing gamma-secretase inhibitors combined with targeted therapies or chemotherapy are currently ongoing (NCT02784795). These trials represents an attractive therapeutic opportunity for a subgroup of patients with this rare, aggressive, and chemo-refractory disease, for which no systemic therapy is currently available.
